# Distribution Law of Corrosion Products in a Marine Chloride Environment

**DOI:** 10.3390/ma15124339

**Published:** 2022-06-19

**Authors:** Jiao Wang, Xinying Ye, Ling Li, Peng Liu

**Affiliations:** 1 Department of Architecture and Civil Engineering, Guangzhou University, Guangzhou 510006, China; wangjiao19890219@sina.com (J.W.); 2111916204@e.gzhu.edu.cn (X.Y.); ll7890808@163.com (L.L.); 2 National Engineering Laboratory for High Speed Railway Construction, 22 Shaoshan Road, Changsha 410004, China

**Keywords:** reinforced concrete, corroded steel bars, corrosion products, radial displacement of concrete, concrete cracking

## Abstract

Steel corrosion is the main cause of reinforced concrete cracking. Conventionally, concrete is considered to crack when the circumferential tensile stress reaches the tensile strength of the concrete. However, few analyses have considered the fracture criteria of the internal cross-section of concrete. Based on the von Mises distribution of angle probabilities, this paper proposes a new probability distribution function for investigating the distribution law of corrosion products. The cracking process of experimental samples was numerically analyzed, and the results were consistent with those of the theoretical model. The effect of the dry–wet cycle ratio on the corrosion products was preliminarily investigated by microscopic observation of the reinforced concrete under different dry–wet cycle corrosion environments.

## 1. Introduction

Reinforcement corrosion increases the volume of corrosion products, causing internal pressure in concrete. Internal pressure leads to concrete cracking, structural damage, and economic losses [1,2,3,4,5]; therefore, investigating concrete cracking caused by steel corrosion is important. Steel corrosion is usually investigated using theoretical and experimental models [6,7,8]. For example, Yuan et al. [9] proposed a distribution rule for the amount of steel corrosion inside the concrete and obtained a distribution curve of the thickness of the rust layer. They also theorized that most of the steel corrosion is distributed on the side facing the concrete cover; thus, the amount of steel corrosion on the side farthest from the cover can be ignored before cracking occurs. However, once the concrete has expanded and cracked, reinforcement corrosion gradually develops by the side near the cover. Yuan et al. [9] further proposed a two-part distribution of the rust layer on the peripheral surface of rebars. The sides closer to and farther from the cover can be modeled using semielliptical and uniform corrosion models, respectively. However, the accuracy of the model remains to be verified. When applying the elasticity theory, Deba et al. [10] simplified the nonuniform rust expansion model of cracks for the concrete cover to a cosine-function rust expansion model. The influences of the reinforcement diameter, concrete strength grade, concrete elastic modulus, and concrete porosity on the corrosion rate of reinforcement were discussed in this model. Xuesong et al. [11] simplified the nonuniform corrosion model with a triangular distribution and studied the expanding corrosion cracks of loaded reinforced concrete beams for 18 months. In the experiments, the circumferential crack width changed exponentially along the radial direction. The corrosion bond strength of the reinforced concrete was then analyzed under the action of the load and environment, and a prediction model of the bond strength of the ribbed steel bars was established. Considering the mechanical properties of rust products and assuming an elliptical distribution of the nonuniform rust expansion force, Liu et al. [12] proposed a formula to express the rust expansion force at the moment of rust expansion cracking.

In a conventional view, the concrete will crack when the circumferential tensile stress reaches the tensile strength of the concrete [13,14,15]. Through experimental observations, Shilang et al. [16] proposed the double-K fracture criterion of concrete. They found that cracks are initiated when the stress intensity factor equals the fracture toughness of the concrete crack initiation. The fracture toughness ($K_{IC}^{ini}$) and instability fracture toughness ($K_{IC}^{un}$) of concrete are both typically measured in fracture experiments on large-scale specimens. However, fracture experiments require measurements of the stable extension length and cracking load before an instability fracture occurs, which are difficult to obtain in laboratory settings [17,18]. Zhimin et al. [19] measured the subcritical expansion displacement of concrete cracks in wedge-splitting tensile specimens; however, their method requires iterative operations. On the basis of the brittle cracking model of concrete, Ping et al. [20] observed the rust expansion cracking of reinforced concrete in a mesonumerical simulation. The crack surface was perpendicular to the direction of the maximum tensile principal stress.

However, the aforementioned studies assumed that the materials exhibited linear elastic tensile behaviors, and did not consider the actual characteristics of the materials. Smarzewski [21] recently investigated how the amount of silica fume affects the mechanical properties of concrete with different water–cement ratios. They found that partially replacing cement with silica fume greatly improves the mechanical properties of high-performance concrete after 28 days; however, high amounts of silica powder increase the brittleness of high-performance concrete. Qing et al. [22] observed a relatively stable crack growth in arc-bent notched specimens. They derived the stress intensity factor K and crack opening displacement by the superposition method. Sadrmomtazi et al. [23] conducted three-point bending tests on 224 slotted beams and analyzed the fracture parameters using the fracture method, size effect method, and boundary effect method. They demonstrated that the water–cement ratio significantly affects the fracture parameters of heavyweight concrete.

The present paper develops a theoretical model of nonuniform corrosion cracking of steel bars. This model is based on a probability distribution function and considers the fracture criteria of the internal cross-section of concrete, which has rarely been considered.

## 2. Distribution of Corrosion Products and Radial Displacement of Reinforced Concrete

### 2.1. Relationship between the Amount of Corrosion and Thickness of the Porous Zone

**Figure 1:**
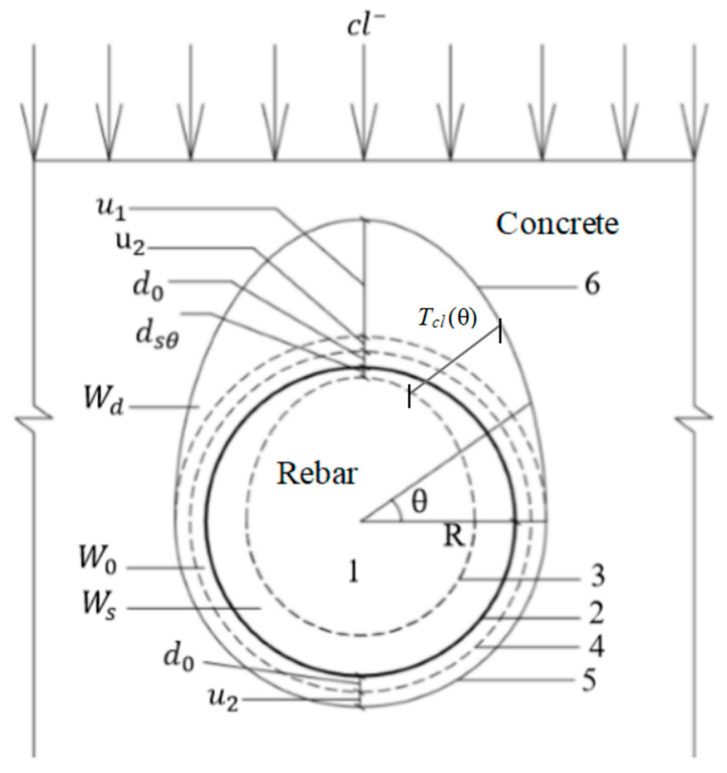
Deformation diagram of the corrosion products of reinforced concrete. 1: reinforcement section; 2: original reinforcement contour; 3: reinforcement corrosion contour; 4: gap contour between reinforced concrete; 5 and 6: thickness contours of the reinforcement corrosion layers on the sides farthest from and closest to the concrete protective layer, respectively.

The corrosion products include the corrosion products filling the void area, in the corroded part of the reinforcement, and beyond the void area (see Figure 1).

In the following, $u_{1}$ and $u_{2}$ represent the unevenly distributed thicknesses of the corrosion products extending beyond the porous zone (mm), where $u_{1}$ denotes the maximum thickness of the corrosion closest to the concrete cover, while $u_{2}$ denotes the thickness of the uniform corrosion at the far side of the concrete cover (mm). The thickness distribution function is denoted as $u_{d}\left(\theta \right)$ (mm). Other variables are defined as follows:

$d_{0}$: the thickness of the void area (mm), distributed as a uniform ring;

$d_{s\theta }$: the consumed thickness of the steel reinforcement (mm), distributed as an ellipse of uneven corrosion;

$W_{0}$: the amount of filled voids (mg);

$W_{d}$: the filling amount of rust products extending beyond the void area (mg);

$W_{s}$: the amount of corrosion filling the consumed part of the reinforcement (mg).

The total amount of corrosion products (mg) is expressed using the following geometric relationship:

(1) $$W_{rust}=W_{0}+W_{s}+W_{d}.$$

In the planar problems, the thickness can be taken as one unit, and the corrosion products filling the voids are given by

(2) $$W_{0}=\rho V_{0}=2\rho \pi Rd_{0}+\rho \pi d_{0}{}^{2},$$

where ρ is the density of the corrosion products (mg/mm^3^), $V_{0}$ is the volume of the corrosion products filling the porous zone (mm^3^), and R is the rebar radius (mm).

According to the geometric relationship in Figure 1, the amount of corrosion in the consumed reinforcement is given by

(3) $$W_{s}=\rho V_{s}=\rho \pi Rd_{s\theta },$$

where $V_{s}$ is the volume of the corrosion products filling the reinforcement (mm^3^).

Beyond the void area, the amount of rust products is calculated as:

(4) $$W_{d}=\rho V_{d}=W_{1}+W_{2}=\frac{\rho \pi }{2}\left(u_{2}+3u_{2}R+3u_{2}d_{0}+u_{1}R+u_{1}d_{0}+u_{1}u_{2}\right),$$

*W*_1_—is the filling amount of the rust products extending beyond the void area corresponding to $u_{1}$.

*W*_2_—is the filling amount of the rust products extending beyond the void area corresponding to $u_{2}$.

*V_d_* is the volume of the corrosion products outside the filling void area (mm^3^).

Substituting Equations (2)–(4) into Equation (1), the total amount of the corrosion products is obtained as:

(5) $$W_{rust}=W_{0}+W_{s}+W_{d}=\rho \pi \left[2d_{0}\left(R+d_{0}\right)+Rd_{s\theta }+\frac{u_{2}\left(1+3R+3d_{0}\right)+u_{1}\left(R+d_{0}\right)+u_{1}u_{2}}{2}\right].$$

Some scientists have demonstrated that the thickness of the void zone $d_{0}$ at the reinforced concrete interface and the uniform corrosion thickness $u_{2}$ at the far side of the concrete cover are minimum values. Therefore, the higher-order infinitesimal terms $u_{2}$, $d_{0}{}^{2}$, and $u_{2}d_{0}$ in Equation (5) can be omitted from the calculation. If the loss thickness of the reinforcement is sufficiently smaller than the diameter of the reinforcement, then $u_{1}$ is the minimum value, and the higher-order infinitesimal terms $u_{1}u_{2}$ and $u_{1}d_{0}$ can also be omitted [24]. Accordingly, the total amount of corrosion products simplifies to

(6) $$W_{rust}=W_{0}+W_{s}+W_{d}=\rho \pi \left[2d_{0}R+Rd_{s\theta }+\frac{3u_{2}R+u_{1}R}{2}\right].$$

### 2.2. Corrosion Rate of Reinforcement upon Cracking of the Concrete Cover

The volume ratio of all the corrosion products to the reinforcement consumed is called the expansion ratio *n* of the corrosion products. Using this ratio, the total volume of the corrosion products (mm^3^) can be related to the volume versus the reinforcement consumed as follows:

(7) $$\;V_{r}=nV_{s}$$

The total volume of the corrosion products is the sum of three parts:

(8) $$V_{r}=V_{0}+V_{s}+V_{d}.$$

In the planar problems, the filling volume of the corrosion products can be obtained by calculating the per-unit thickness as follows:

(9) $$V_{0}=2\pi Rd_{0},$$

(10) $$V_{d}=\frac{\left(3u_{2}R+u_{1}R\right)\pi }{2}.$$

Combining Equations (7) and (8), the total volume of the corrosion products is given by

(11) $$V_{r}=V_{0}+V_{s}+V_{d}=nV_{s}.$$

Substituting Equations (9) and (10) into Equation (11) yields

(12) $$2\pi Rd_{0}+V_{s}+\frac{\left(3u_{2}R+u_{1}R\right)\pi }{2}=nV_{s}.$$

After simplification, the volume $V_{s}$ of the rusted part of the reinforcement is obtained as follows:

(13) $$V_{s}=\frac{4\pi Rd_{0}+\pi R\left(u_{1}+3u_{2}\right)}{2\left(n-1\right)}.$$

From Equation (13), the corrosion rate is obtained as follows:

(14) $$\rho =\frac{m_{s}}{m_{sr}}\times 100\%=\frac{V_{s}}{V_{sr}}\times 100\%=\frac{4d_{0}+u_{1}+3u_{2}}{2\left(n-1\right)R}\times 100,$$

where $m_{s}$ is the mass consumed by the reinforcement (mg), $m_{sr}$ is the mass of the original reinforcement (mg), $V_{s}$ is the volume consumed by the reinforcement (mm^3^), and $V_{sr}$ is the volume of the original reinforcement (mm^3^).

### 2.3. Distribution of Corrosion Products of Steel Bars

Zhao et al. and Wong et al. [25,26,27] studied the distributions of corrosion products. Through long-term corrosion tests in an artificial environment, they demonstrated that the corrosion thickness can be described using a Gaussian function as follows:

(15) $$T_{cl}(\theta )=\frac{\alpha_{1}}{\alpha_{2}\sqrt{2}\pi }e^{-{\left(\frac{\theta -\mu }{\sqrt{2}\alpha_{2}}\right)}^{2}}+\alpha_{3},$$

where $T_{cl}\left(\theta \right)$ is the total thickness of the rust layer (mm), μ is the maximum thickness of the rust products, (which can be set to π), $\alpha_{1}$ is the nonuniformity coefficient of the rust layer, and $\alpha_{2}$ and $\alpha_{3}$ are the spread and uniformity coefficients of the rust layer, respectively.

The general Gaussian distribution function is given by

(16) $$f\left(x\right)=\frac{1}{\sigma \sqrt{2}\pi }e^{-{\left(\frac{x-\xi }{\sqrt{2}\sigma }\right)}^{2}},$$

where ξ and σ are the mean and standard deviation of the random variables, respectively.

Notably, the Gaussian function of the corrosion thickness given by Equation (15) has the same form as the general Gaussian distribution function given by Equation (16). The von Mises distribution function [28] models a continuous probability distribution on a circle, which closely fits the Gaussian distribution function. In other words, the von Mises distribution function can well describe the uneven distributions of the corrosion products of steel bars. To characterize the distribution of the corrosion products of steel bars, one can use the revised von Mises distribution function [29] given by

(17) $$T_{cl}\left(\theta \right)=\delta \frac{e^{kcos\left(\theta -\mu \right)}}{2\pi I_{0}\left(k\right)},$$

where $I_{0}$ is the modified zero-order Bayesian function, *k* is the nonuniformity coefficient, μ is the distribution peak, and δ is the correction factor.

$T_{cl}\left(\theta \right)$ is related to $d_{s\theta }$ as follows:

(18) $$T_{cl}(\theta )=nd_{s\theta }$$

The total thickness of the corrosion products is obtained by summing the consumed thickness of the steel reinforcement, the void-area thickness and the thickness outside the void area, as illustrated in Figure 1. Therefore, the total thickness of the rust layer is given as:

(19) $$T_{cl}\left(\theta \right)=d_{s\theta }+d_{0}+u_{d}\left(\theta \right).$$

where $u_{d}\left(\theta \right)$ is the thickness distribution function that denotes the thickness outside the void area.

Combining Equations (18) and (19), the relationship between $d_{s\theta }$ and $u_{d}\left(\theta \right)$ is given as:

$$(n-1)d_{s\theta }=d_{0}+u_{d}(\theta ),$$

(20) $$d_{s\theta }=\frac{1}{n-1}d_{0}+\frac{1}{n-1}u_{d}(\theta )$$

Substituting Equation (20) into Equation (19), the total thickness of the corrosion products is given by

(21) $$T_{cl}\left(\theta \right)=\frac{1}{n-1}d_{0}+\frac{1}{n-1}u_{d}\left(\theta \right)+d_{0}+u_{d}\left(\theta \right)=\frac{n}{n-1}d_{0}+\frac{n}{n-1}u_{d}\left(\theta \right)$$

Simplifying Equation (21), the thickness outside the void area is calculated as:

$$\frac{n}{n-1}u_{d}\left(\theta \right)=T_{cl}\left(\theta \right)-\frac{n}{n-1}d_{0},$$

(22) $$u_{d}\left(\theta \right)=\frac{n-1}{n}T_{cl}\left(\theta \right)-d_{0}.$$

In the planar problems, the volume of the corrosion products outside the filling void area $V_{d}$ is calculated by integrating the unit thickness:

(23) $$V_{d}=\frac{1}{2}\int_{0}^{2\pi }\left(u_{d}\left(\theta \right)+d_{0}+R\right)d\theta -\pi {\left(R+d_{0}\right)}^{2}=\frac{1}{2}\int_{0}^{2\pi }u_{d}{\left(\theta \right)}^{2}+2u_{d}\left(\theta \right)\left(d_{0}+R\right)d$$

When the thickness loss of the steel bars is sufficiently smaller than the diameter of the steel bars, $u_{d}\left(\theta \right)$ can be regarded as the minimum value, so the higher-order infinitesimals $u_{d}{\left(\theta \right)}^{2}$ and $u_{d}(\theta )d_{0}$ in Equation (23) can be omitted. Therefore, $V_{d}$ simplifies to

(24) $$\begin{array}{ll} V_{d} & =\int_{0}^{2\pi }u_{d}\left(\theta \right)Rd\theta \\ & =\frac{n-1}{n}R\;\delta \int_{0}^{2\pi }\frac{e^{kcos\left(\theta -\mu \right)}}{2\pi I_{0}\left(k\right)}d\theta -2\pi d_{0}R. \end{array}$$

In Equation (24), the integral term $\int_{0}^{2\pi }\frac{e^{kcos\left(\theta -\mu \right)}}{2\pi I_{0}\left(k\right)}d\theta$ represents the probability of the von Mises distribution function on the entire circle. If its value is 1, Equation (24) further simplifies to

(25) $$V_{d}=\frac{n-1}{n}R\;\delta -2\pi d_{0}R.$$

From Equations (9) and (11), the following equation is obtained:

(26) $$V_{r}=V_{0}+V_{s}+V_{d}=nV_{s}=\frac{n}{n-1}V_{d}+\frac{n}{n-1}\cdot 2\pi d_{0}R.$$

When substituting Equation (25) into Equation (26), the total volume of the corrosion products $V_{r}$ can be expressed as follows:

(27) $$V_{r}=\frac{n}{n-1}V_{d}+\frac{n}{n-1}\cdot 2\pi d_{0}R=R\;\delta .$$

From Equation (27), the correction coefficient δ is obtained as:

(28) $$\delta =\frac{V_{r}}{R}.$$

Substituting Equation (28) into Equation (17), the total thickness of the corrosion products is calculated as:

(29) $$T_{cl}\left(\theta \right)=\frac{V_{r}}{R}\frac{e^{kcos\left(\theta -\mu \right)}}{2\pi I_{0}\left(k\right)}.$$

From Equation (14), the reinforcement corrosion rate is given by

(30) $$\rho =\frac{m_{s}}{m_{sr}}=\frac{V_{s}}{V_{sr}}=\frac{V_{s}}{\pi R^{2}}.$$

Substituting Equation (30) into Equation (29) yields the following relationship between the total thickness of the corrosion products and the corrosion rate of the steel bars:

(31) $$T_{cl}\left(\theta \right)=\frac{V_{r}}{R}\frac{e^{kcos\left(\theta -\mu \right)}}{2\pi I_{0}\left(k\right)}=n\rho \pi R\frac{e^{kcos\left(\theta -\mu \right)}}{2\pi I_{0}\left(k\right)}.$$

When substituting Equation (31) into Equation (22), the thickness outside the void area $u_{d}\left(\theta \right)$ can be expressed as follows:

(32) $$u_{d}\left(\theta \right)=\frac{n-1}{n}T_{cl}\left(\theta \right)-d_{0}=\left(n-1\right)\rho \pi R\frac{e^{kcos\left(\theta -\mu \right)}}{2\pi I_{0}\left(k\right)}-d_{0}.$$

From Equation (32), the maximum filling thickness of the corrosion products is given as:

(33) $$u_{d}(\theta )=(n-1)\rho R\frac{1}{2I_{0}(k)}-d_{0}$$

### 2.4. Radial Displacement of Corroded Reinforced Concrete

**Figure 2:**
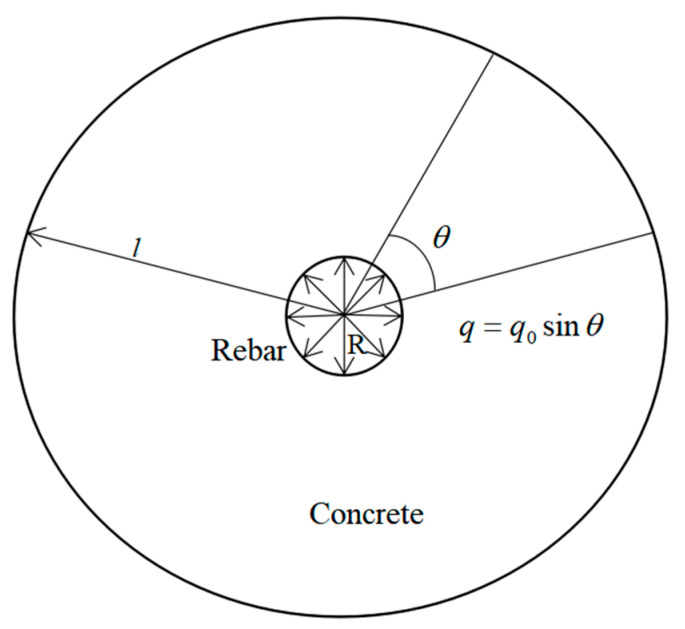
Action of corrosion stress in reinforced concrete.

The thickness distribution of the rust layer is assumed to be a semi-ellipse along the upper half-circle, which can be approximately represented using a sine function (see Figure 2). As the rust expansion force of the steel bars is proportional to the thickness of the rust layer, it can also be approximated using a sine function, namely, *q* = *q*_0_*sinθ*, where $q_{0}$ is the maximum reinforcement expansion force corresponding to θ = *π*/*2*. Additionally, as the boundary stress changes with θ, the stress function is assumed to be φ=φ(r,θ), where (r,θ) represents any point on the circle. In elastic mechanics theory, the stress function of a section of the test specimens is expressed as:

(34) $$\varphi =\left(A\ln r+Br^{2}\right)sin\theta ,$$

where *A* and *B* are the coefficients determined by the boundary conditions and constraints.

*R* is the radius of the steel bars (mm), and *l* is the maximum radius of the ring (mm).

In the elastic mechanics theory, the stress component can be represented by the radial normal, circumferential normal, and shear stresses in the plane, respectively, given by

$$\sigma_{r}=\frac{1}{r}\cdot \frac{\partial \varphi }{\partial r}+\frac{1}{r^{2}}\cdot \frac{\partial^{2}\varphi }{\partial \theta^{2}}=\left[\frac{A}{r^{2}}\left(1-\ln r\right)+B\right]\;sin\theta$$

(35) $$\sigma_{\theta }=\frac{\partial^{2}\varphi }{\partial r^{2}}=\left(-\frac{A}{r^{2}}+2B\right)sin\theta$$

$$\tau_{r\theta }=-\frac{\partial }{\partial r}\left(\frac{1}{r}\cdot \frac{\partial \varphi }{\partial \theta }\right)=-\left[\frac{A}{r^{2}}\left(1-\ln r\right)+B\right]\;\cos\theta .$$

When substituting the boundary condition into Equation (35), the coefficients *A* and *B* are obtained as follows:

$$\sigma_{r}{|}_{\theta =\frac{\pi }{2};\;r=l}=0;\;\tau_{r\theta }{|}_{\theta =\frac{\pi }{2};\;r=l}=0;\;\sigma_{r}{|}_{\theta =\frac{\pi }{2};\;r=R}=-q_{0}sin\theta =-q_{0};$$

(36) $$\{\begin{array}{c} \frac{A}{l^{2}}\left(1-\ln l+B\right)=0;\\ -\left[\frac{A}{l^{2}}\left(1-\ln l\right)+B\right]\;\cos\theta =0;\Rightarrow \\ \frac{A}{R^{2}}\left(1-\ln R+B\right)=-q_{0}; \end{array}\{\begin{array}{c} A=-\frac{R^{2}l^{2}q_{0}}{l^{2}\left(1-\ln R\right)-R^{2}\left(1-\ln l\right)}\\ B=\frac{\left(1-\ln l\right)R^{2}q_{0}}{l^{2}\left(1-\ln R\right)-R^{2}\left(1-\ln l\right)} \end{array}$$

From Equations (35) and (36), the hoop stress at $\theta =\frac{\pi }{2}$ is determined as:

(37) $$\{\begin{array}{c} \sigma_{\theta }{|}_{\theta =\frac{\pi }{2};\;r=R}=-\frac{A}{R^{2}}+2B=\frac{l^{2}+2R^{2}\left(1-\ln l\right)}{l^{2}\left(1-\ln R\right)-R^{2}\left(1-\ln l\right)}q_{0}\;\\ \sigma_{\theta }{|}_{\theta =\frac{\pi }{2};\;r=l}=-\frac{A}{l^{2}}+2B=\frac{R^{2}\left(3-\ln l\right)}{l^{2}\left(1-\ln R\right)-R^{2}\left(1-\ln l\right)}q_{0} \end{array}$$

When the maximum tensile stress at the interface of the reinforced concrete $\sigma_{\theta }$ exceeds the ultimate tensile strength of concrete $f_{t}$, the concrete will crack; that is,

(38) $$\sigma_{\theta }{|}_{\theta =\frac{\pi }{2};\;r=R}=f_{t}.\;$$

When substituting Equation (38) into Equation (37), the maximum rust expansion force is given by

(39) $$q_{0}=\frac{l^{2}\left(1-\ln R\right)-R^{2}\left(1-\ln l\right)}{l^{2}+2R^{2}\left(1-\ln l\right)}f_{t}.\;$$

By substituting Equation (39) into Equation (36), the coefficients *A* and *B* at the critical cracking state are obtained as:

(40) $$A=-\frac{R^{2}l^{2}}{l^{2}\left(1-\ln R\right)-R^{2}\left(1-\ln l\right)}\cdot \frac{l^{2}\left(1-\ln R\right)-R^{2}\left(1-\ln l\right)}{l^{2}+2R^{2}\left(1-\ln l\right)}f_{t}=-\frac{R^{2}l^{2}}{l^{2}+2R^{2}\left(1-\ln l\right)}f_{t},\;\;=\frac{\left(1-\ln l\right)R^{2}}{l^{2}\left(1-\ln R\right)-R^{2}\left(1-\ln l\right)}\cdot \frac{l^{2}\left(1-\ln R\right)-R^{2}\left(1-\ln l\right)}{l^{2}+2R^{2}\left(1-\ln l\right)}f_{t}=\frac{R^{2}\left(1-\ln l\right)}{l^{2}+2R^{2}\left(1-\ln l\right)}f_{t}.\;$$

The radial strain is obtained using the following physical equation:

(41) $$\varepsilon_{r}=\frac{1}{E}\left(\sigma_{r}-\upsilon \sigma_{\theta }\right)=\frac{1}{E}\left[\frac{A}{r^{2}}\left(1-\ln r\right)+B-\upsilon \left(-\frac{A}{r^{2}}+2B\right)\right]sin\theta .$$

The radial strain is related to the radial displacement by the geometric equation

(42) $$\varepsilon_{r}=\frac{\partial u_{r}}{\partial r}.$$

When substituting Equation (41) into Equation (42), the radial displacement is given as:

(43) $$u_{r}=\frac{1}{E}\left[\frac{A\ln r}{r^{2}}-\frac{A\upsilon }{r}+\left(1-2\upsilon \right)Br\right]sin\theta .$$

Finally, when substituting Equation (40) into Equation (43), the maximum radial displacement of concrete at the critical state of interface cracking (i.e., r=R) is obtained as follows:

(44) $$\begin{array}{l} u_{rmax}=\frac{1}{E}\left[-\frac{R^{2}l^{2}}{l^{2}+2R^{2}\left(1-\ln l\right)}f_{t}\cdot \left(\frac{\ln R}{R^{2}}-\frac{\upsilon }{R}\right)+\frac{R^{2}\left(1-\ln l\right)}{l^{2}+2R^{2}\left(1-\ln l\right)}f_{t}\;\left(1-2\upsilon \right)R\right]sin\theta \\ \;=\frac{1}{E}\cdot \frac{R^{2}f_{t}}{l^{2}+2R^{2}\left(1-\ln l\right)}\left[\left(1-\ln l\right)\left(1-2\upsilon \right)R-l^{2}\left(\frac{\ln R}{R^{2}}-\frac{\upsilon }{R}\right)\right], \end{array}$$

where E denotes the modulus of elasticity of concrete (MPa) and υ is the Poisson ratio of concrete.

When combining Equations (33) and (44), the limit state equation of concrete cracking is expressed as:

(45) $$Z\left(k\right)=u_{rmax}-u_{d}\left(\theta \right).\;$$

## 3. Reliability Analysis of Concrete Cracking

In the limit state equation proposed in Section 2.4, the radial displacement of concrete and the filling thickness of the corrosion products are regarded as the resistance and load effect, respectively. In this study, the reliability of concrete cracking was analyzed using a second-moment algorithm. Moreover, during the chloride corrosion process, the nonuniformity coefficient of the corrosion products around the reinforcement will vary. Therefore, this paper investigated the reliability of different distributions of corrosion products.

Setting *R = u_rmax_* and *S = u_d_(θ)*, the performance function of concrete cracking described using Equation (45) is newly defined as

(46) Z(k)=g(R,S)=R−S.

If different nonuniformity coefficients have different effects on corrosion cracking, the corresponding reliability index can be expressed as follows:

(47) $$\beta =\frac{\mu_{R}-\mu_{S}}{\sqrt{\sigma_{R}^{2}+\sigma_{S}^{2}}},\;$$

where $\mu_{R}\;(\sigma_{R}$) and $\mu_{S}$ ($\sigma_{S}$) are the means (standard deviations) of the random variables *R* and *S*, respectively (in mm).

The averages and standard deviations of the concrete compressive strength *f*_c_, cover thickness *c*, reinforcement diameter *d*, and corrosion rate *ρ* in the existing data [30] are provided in Table 1.

**Figure 3:**
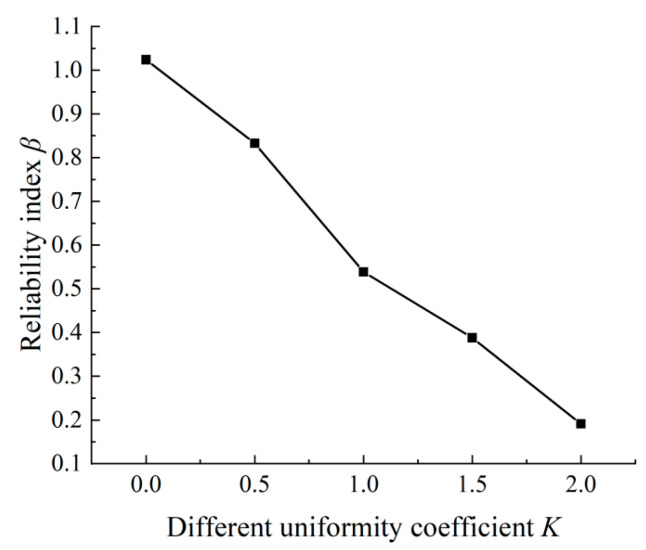
Reliability index of the analysis versus nonuniformity coefficient *K*.

From the statistical data in the relevant literature [31,32,33], the reliability index *β* for the different nonuniformity coefficients was calculated using the aforementioned formulas, and the results are plotted in Figure 3. The larger the value of the nonuniformity coefficient *K*, the smaller the *β*. This trend is reasonable because *K* represents the filling degree of the rust products around the reinforcement; therefore, as *K* increases, the rust products become more concentrated. When 0 ≤ *K* ≤ 1, the rate of the reliability index was at the maximum, indicating a clear influence of the nonuniformity coefficient on the reliability index during the filling process. When *K* exceeded 1, the influence of the nonuniformity coefficient on the reliability index was reduced.

## 4. Experimental Section

### 4.1. Preparation of Materials and Specimens

To improve the accuracy of the experimental results, no active agent, except the sodium chloride solution, was added when pouring the concrete. After trial-and-error and adjustments of the mix proportion following the relevant methods specified by the Regulation of Common Concrete Mix Design (JGJ55-2011), the final concrete mix for the test was determined (see Table 2).

**Figure 4:**
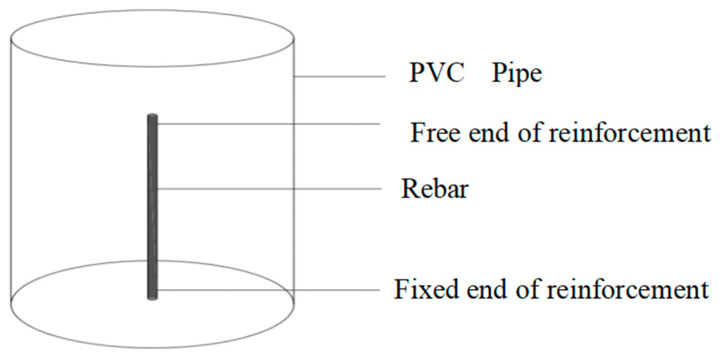
Schematic of the reinforced concrete samples (PVC = polyvinyl chloride).

Figure 4 presents the configuration of the concrete specimens and the layout of the rebars. The specimens were prepared with the same position of reinforcement, but with different concrete-cover thicknesses and reinforcement diameters. After curing the 48 samples in the natural environment for 28 days, a 7% NaCl solution was added.

### 4.2. Corrosion Acceleration

After 28 days of moist curing, the specimens were exposed to an artificial environment to accelerate the steel corrosion. The artificial environment was introduced in two stages: corrosion initiation and corrosion propagation. Corrosion initiation involved exposing the specimens to a cyclic dry–wet environment for 1–3 months. Each dry–wet cycle included wetting in a 7% (by mass) NaCl solution and oven-drying at 65 °C (total time = 1 day per cycle). To ensure a similar amount of chloride around the surface of each cross-section, each specimen was placed vertically in a container. The details of the sample groups are provided in Table 3.

### 4.3. Experimental Results and Analysis

The cracking process of concrete through dry–wet cycling is a long-term process, requiring over 1–3 months. The cracking times of the samples are listed in Table 4.

- Influence of the thickness of the concrete cover on the cracking time

**Figure 5:**
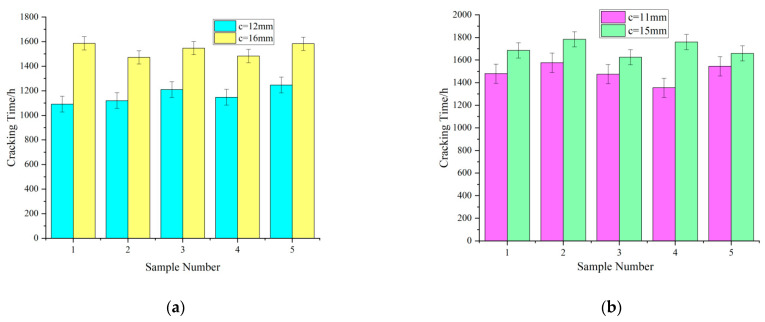
Effect of concrete cover thickness on cracking time. (**a**) Cracking times of samples with different concrete-cover thicknesses (*d* = 8 mm), (**b**) Cracking times of samples with different concrete covers (*d* = 10 mm).

In the specimens with the same reinforcement diameter and concrete strength, the cracking time of the specimens increased with the increasing thickness of the concrete cover (Figure 5). As the thickness of the concrete cover increased, the penetration path of the external corrosive medium (the chloride ions and corrosive medium in the solution) increased, increasing the corrosion time of the reinforcement.

- Influence of the reinforcement diameter on the cracking time

**Figure 6:**
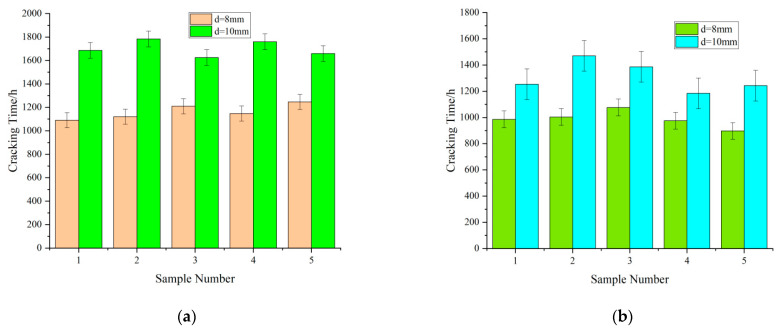
Effect of reinforcement diameter on cracking time. (**a**) Cracking times of samples with different reinforcement diameters *d* (*c* ≈ 15 mm); (**b**) cracking times of samples with different reinforcement diameters *d* (*c* ≈ 12 mm).

As illustrated in Figure 6, the increasing reinforcement diameter lengthened the cracking time of the concrete cover. For a constant amount of corrosion, the rust expansion force of the corrosion products was more dispersed into the surrounding concrete in the samples with larger reinforcement diameters than in the samples with smaller reinforcement diameters.

- Effect of the ratio of dry–wet cycles on the cracking time

**Figure 7:**
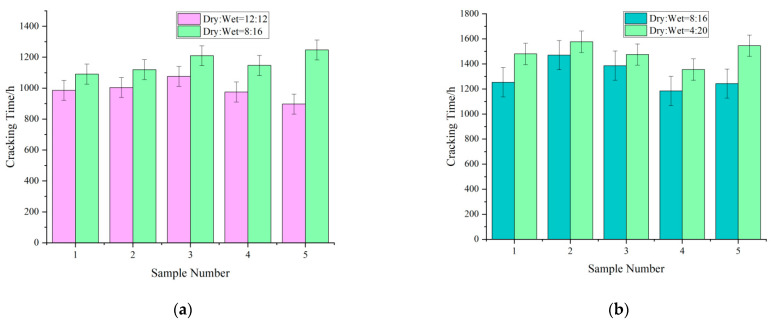

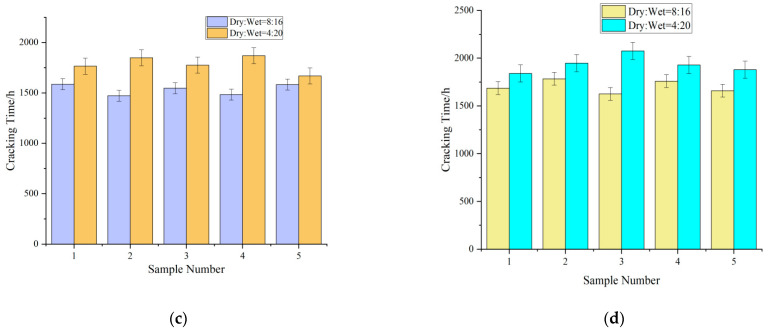
Effect of drying–wetting cycles on cracking time of the specimens. (**a**) Cracking time of specimens in different drying–wetting cycles (*l* = 32 mm, *d* = 8 mm); (**b**) cracking time of specimens in different drying–wetting cycles (*l* = 32 mm, *d* = 10 mm); (**c**) cracking times of specimens in different drying–wetting cycles (*l* = 40 mm, *d* = 8 mm); (**d**) cracking times of specimens in different drying–wetting cycles (*l* = 40 mm, *d* = 10 mm).

As illustrated in Figure 7, reducing the drying time increased the cracking time of the concrete cover in specimens with the same rebar diameter in the same cycle. This trend can be explained by the high-temperature environment used for accelerated drying, which facilitated the entry of chloride ions into the concrete.

## 5. Macroscopic Characteristics of Samples under an Optical Microscope

### 5.1. Distribution of Corrosion Products in Cross-Sections of the Samples

The specimens were observed using optical microscopy (OM), environmental scanning electron microscopy (SEM), energy-dispersive spectrometry, and X-ray diffraction (XRD). SEM only observes the surface morphology of the rust layer and cannot depict the true color of the material. Therefore, the sections of reinforced concrete were observed using both the environmental SEM and OM. For convenience, we inserted the test block along the crack with a slotted screwdriver and then pried the test block to remove the inner rust layer. To avoid further oxidation in the air, the test block was quickly transferred to the X-ray diffractometer for observation.

**Figure 8:**
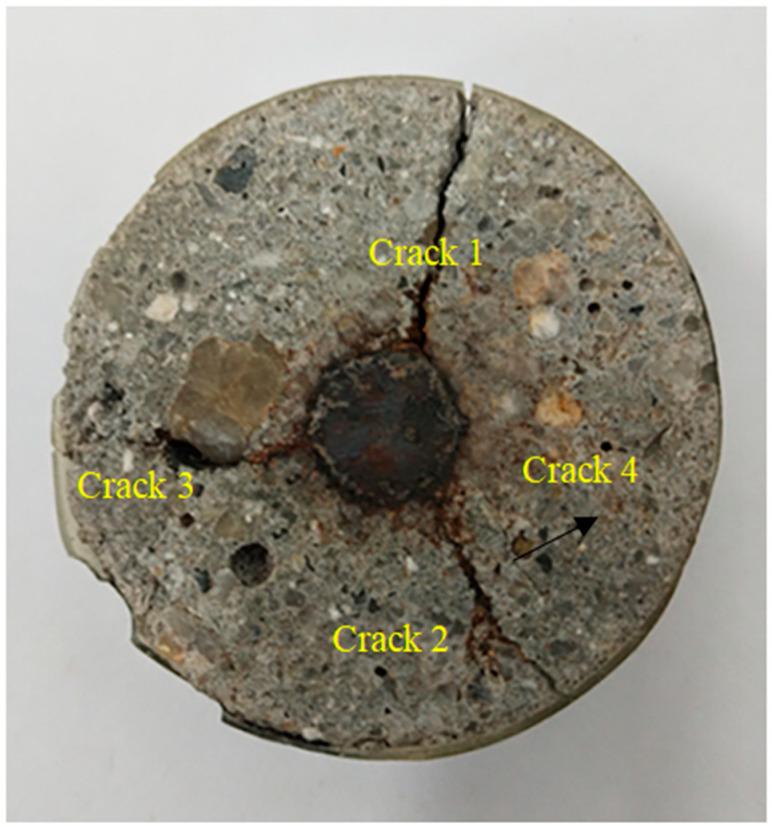
Reinforced concrete section.

**Figure 9:**
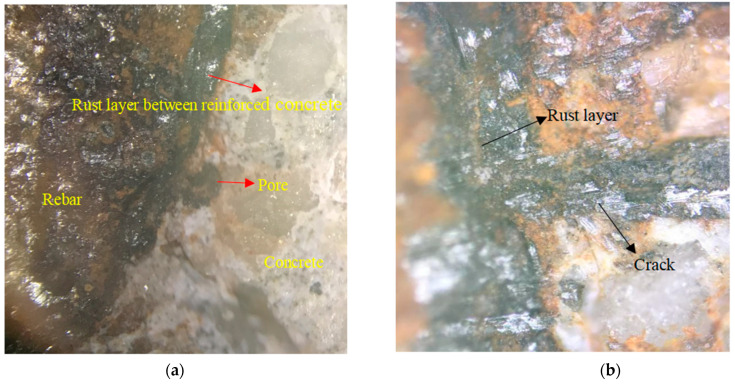
Distribution of corrosion products in a reinforced concrete section. (**a**) Corrosion products of the concrete interface and pore; (**b**) corrosion products of reinforced concrete interface and cracks.

The distributions of the corrosion products in the cracks and interfaces were observed and analyzed using OM. Figure 8 displays a sample with three main cracks and several scattered internal cracks. The cracks, labeled 1, 2, and 3, are external cracks, while crack 4 is an internal crack. The coarse aggregate notably affected the development of crack 3, causing a sudden increase in its crack width. Figure 9 shows the distribution of the corrosion products obtained using OM. Figure 9a,b indicates that the reinforced concrete interface was a black rust layer. This layer formed because the expansion force of the corrosion products acted on the surrounding concrete and the corrosion products themselves were squeezed by the surrounding concrete. As shown in Figure 9a, some of the pores were dark gray in the center, indicating that the rust products were not full of pores. Furthermore, the pore interior was black and the rust products were reddish-brown rust, indicating a small amount of air in the pores that further oxidized the corrosion products. Figure 9b is an enlarged view of crack 1. The cracks close to and far from the rust layer were filled with black and reddish-brown rust products, respectively. This observation can be explained by the complete oxidation of the corrosion products in the outer cracks, which were exposed to the natural environment. Additionally, the low oxygen content near the reinforcement likely suppressed further oxidation of the corrosion products.

### 5.2. Rust Pits on the Surface of Corroded Reinforcement

**Figure 10:**
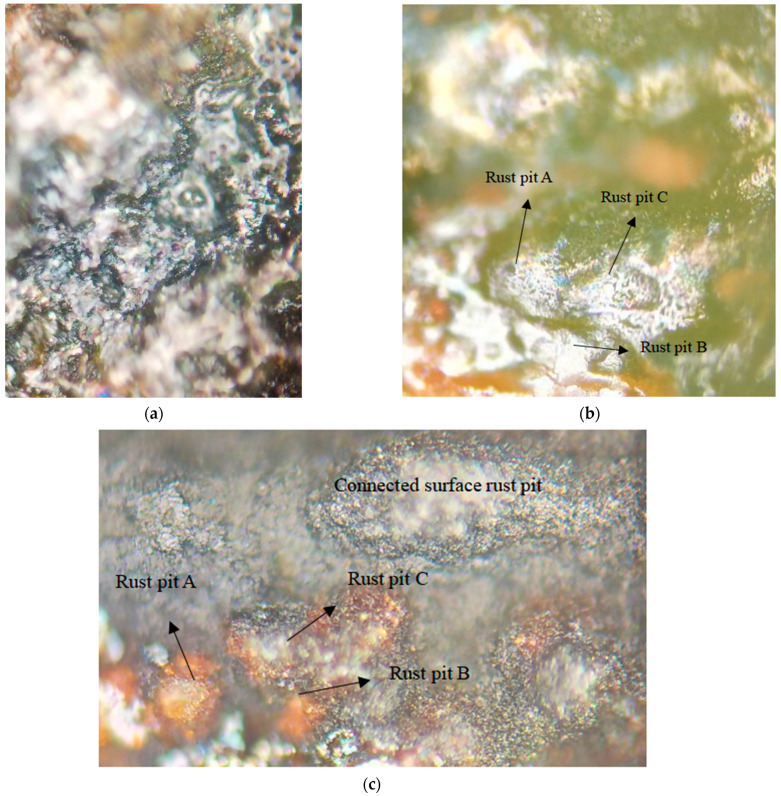
Rusted surface of the reinforcement. (**a**) Distribution of rust pits on the surface; (**b**) adjacent rust pits on the reinforcement surface; (**c**) development of rust pits on the surface of steel bars.

Figure 10 displays the surface morphology of the reinforcement in the wet–dry cycle environment. As shown in Figure 10a, many rust pits of different sizes and depths developed on the surface of the reinforcement. The adjacent rust pits were gradually connected and became shallow in their depth (Figure 10b). After further development (Figure 10c), the adjacent rust pits had connected into short rods with an obviously shallow depth and enlarged width.

**Figure 11:**
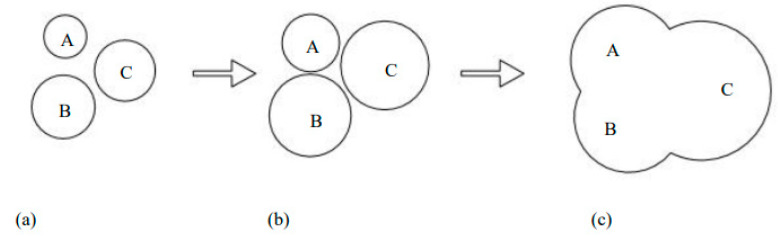
Development mode of rust pits A, B and C on the surface of steel bars. (**a**) Various sites of rust pits on the reinforcement surface; (**b**) rust pits developed toward the surrounding areas; (**c**) a large shallow pit and large rust pit.

The development mode of the rust pits on the reinforcement surface in the dry–wet cycle environment, inferred from the aforementioned analysis, is illustrated in Figure 11. In the chloride corrosion environment, pitting corrosion occurred at various sites on the reinforcement surface (Figure 11a). Rust pits developed; however, not toward the radius of the reinforcement, but toward the surrounding areas of the rust pit (Figure 11b). Finally, the adjacent rust pits thus connected to form a large shallow pit and large rust pit (Figure 11c). Because the rust products accumulated in the rust pits and were compacted by the surrounding concrete, water, and oxygen could not easily contact the iron in the depth direction.

### 5.3. Corrosion Products of Reinforced Concrete Interface

**Figure 12:**
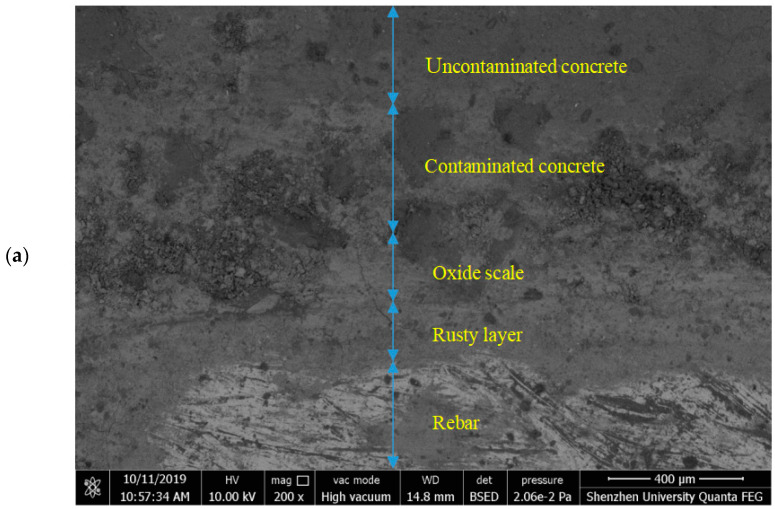

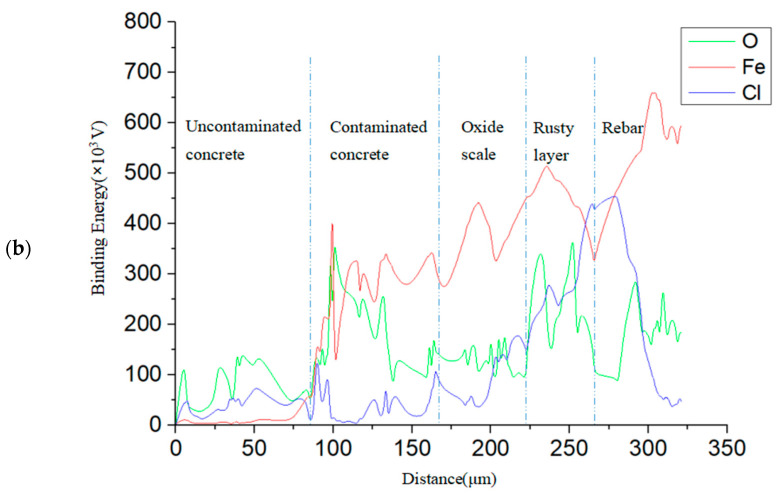
Distribution of corrosion products on the reinforced concrete interface. (**a**) SEM image and energy spectrum analysis line of the reinforced concrete interface, (**b**) binding-energy distribution of elements at the reinforced concrete interface.

Figure 12 shows the energy spectrum derived from the backscattering electron imaging and line analysis at the reinforced concrete interface. The interface of the reinforced concrete exhibited four obvious layers (Figure 12a). From the environmental SEM imaging, the material distribution was inferred as the reinforcement, rust layer, oxide skin, and concrete, which are, respectively, marked in Figure 12a. The aforementioned analysis results were verified using the energy spectrum in Figure 12b.

The energy spectrum in Figure 12b was obtained using quantitative analysis along the line marked in Figure 12a. The spectrum shows the energy distributions of the Fe, O, and Cl elements along the analysis line. The Fe and O distributions along the reinforced concrete interface were clearly divisible into five regions: reinforcement, rust layer, oxide skin, contaminated concrete, and uncontaminated concrete (imaged from the bottom to the top in Figure 12a), and the corrosion products were generated between the reinforcement and the oxide skin. Moreover, the energy of the chloride distribution indicated a high concentration of chloride ions in the corrosion product area, especially on the side closer to the reinforcement. Given that chloride ions are highly permeable, they penetrated the peroxide skin and preferentially oxidized the reinforcement.

### 5.4. Corrosion Products of Concrete Cracks

**Figure 13:**
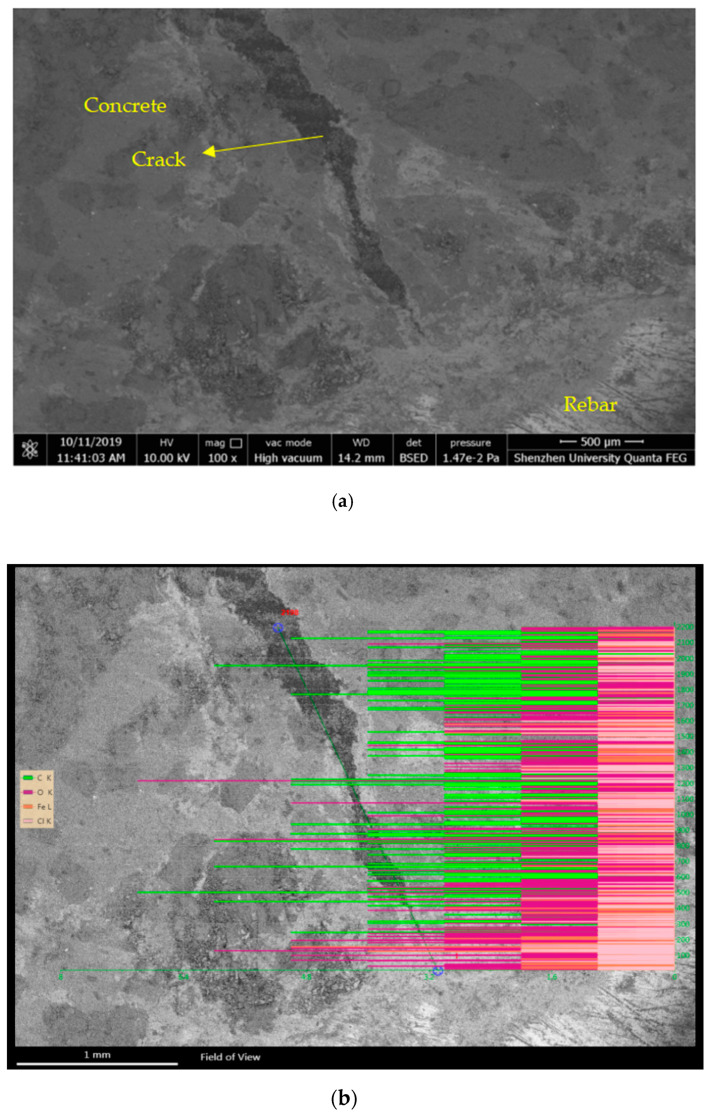
Distribution of corrosion products in a concrete crack. (**a**) SEM image of a concrete crack; (**b**) energy spectrum analysis line and strength distribution of each element in the concrete crack.

Figure 13 shows the energy spectrum and line analysis along the direction of the crack development derived from the backscattering of electron imaging. As illustrated in Figure 13a, the development point of the concrete crack was separated from the reinforcement surface. The corrosion products first filled the pore area between the reinforcement and concrete and then continued to accumulate. Eventually, the weakest area of the concrete reached the ultimate tensile strength. In addition, the crack tip was widened by the relative slipping between the reinforcement and concrete (Figure 13a), and the narrow development of the crack widened when the crack penetrated the concrete. Because the bond belt between the aggregate and the mortar had a low tensile strength, most of the tensile fractures occurred on the bond surface. Figure 13b shows the strength distributions of the four elements in the concrete crack. The strengths are indicated by the lengths of the lines, while the elements are distinguished by the four colors. In general, a denser signal strength denotes a higher element content. Figure 13b reveals that the carbon content decreased with an increasing depth. Papadakis et al. [34] considered that when the relative humidity exceeds 70%, the carbon dioxide concentration decreases linearly with the depth in concrete pores.

As shown in Figure 13b, the chloride signal was strongest at the crack tip, indicating the high permeability of chloride ions. When entering concrete, the chloride ions destroy the passive film on the surface of the reinforcement, forming a corrosion battery that further erodes the reinforcement.

### 5.5. Composition of Rust Products Studied Using X-ray Diffraction

**Figure 14:**
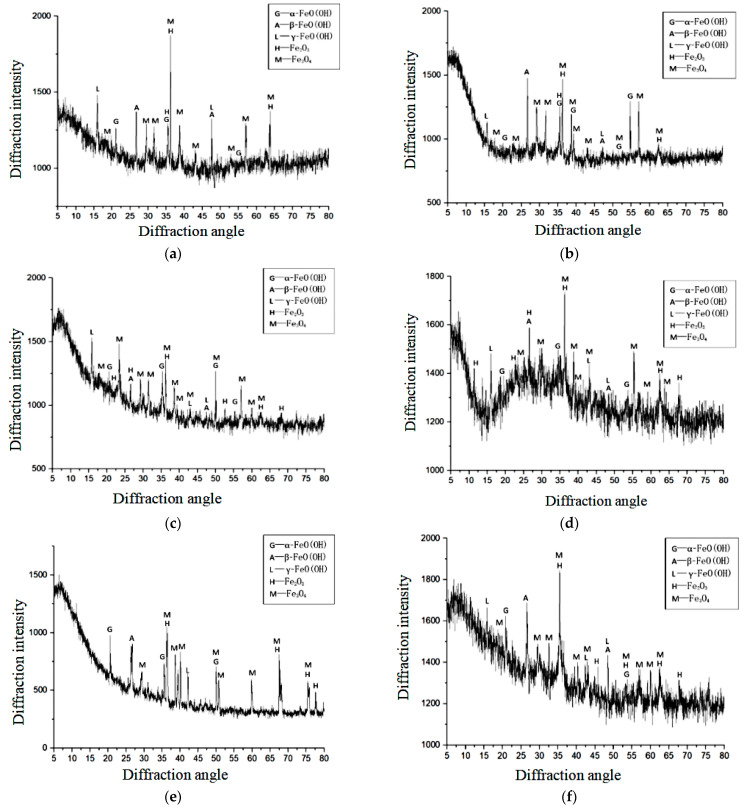

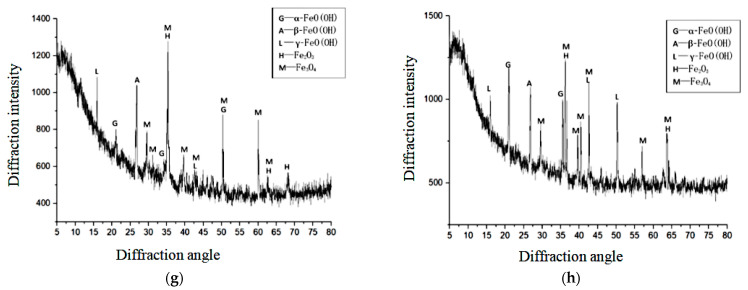
X-ray diffraction results of the corrosion products in different specimens. (**a**) X-ray diffraction pattern (*d* = 10 mm, *l* = 32 mm, dry:wet = 12:12), (**b**) X-ray diffraction pattern (*d* = 10 mm: *l* = 32 mm, dry:wet = 8:16), (**c**) X-ray diffraction pattern (*d* = 8 mm, *l* = 32 mm, dry:wet = 12:12), (**d**) X-ray diffraction pattern (*d* = 8 mm, *l* = 40 mm, dry:wet = 8:16), (**e**) X-ray diffraction pattern (*d* = 8 mm, *l* = 32 mm, dry:wet = 8:16), (**f**) X-ray diffraction pattern (*d* = 8 mm, *l* = 40 mm, dry:wet = 4:20), (**g**) X-ray diffraction pattern (*d* = 10 mm, *l* = 40 mm, dry:wet = 8:16), (**h**) X-ray diffraction pattern (*d* = 10 mm, *l* = 40 mm, dry:wet = 4:20).

The XRD patterns of eight samples in this experiment were compared with the standard XRD pattern of iron oxide. The results are displayed in Figure 14.

The peaks attributable to the corrosion products appeared at the same positions in the diffraction patterns of each specimen, and the strongest peak appeared at approximately θ = 36° (where θ is the diffraction angle). Although the heights of the diffraction peaks of the corrosion products differed among the specimens, the corrosion products were clearly very similar in each specimen.

The reinforcement in concrete actually corrodes via an electrochemical process [35,36,37,38,39]. The high alkalinity of cement hydration products is known to passivate the surface of the reinforcement and then form a dense passivation film. However, corrosion caused by chloride ions reduces the pH value and disrupts the passivation film. At this time, the steel bar is in the activated state.

**Figure 15:**
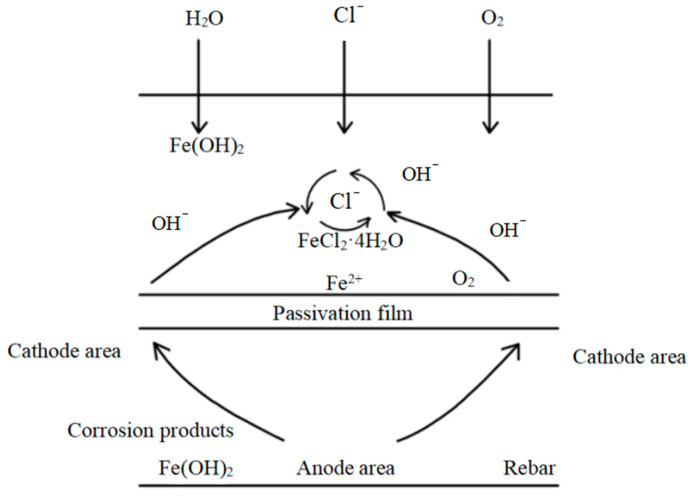
Corrosion diagram of reinforced concrete.

Corrosion proceeds in four basic stages [40], as depicted in Figure 15.

From the XRD pattern, the major corrosion products in each specimen were discerned as α-FeO(OH), β-FeO(OH), γ-FeO(OH), Fe_2_O_3_, and Fe_3_O_4_. Among these products, γ-FeO(OH) is a transitional hydroxyl iron oxide that assists the formation of the most stable hydroxyl iron oxide, α-FeO(OH). When concrete exists in a long-term immersion state, its oxygen supply is limited. On the contrary, when the concrete dries over a long period of time, its interior is relatively dry and the oxygen supply is abundant. During the dry–wet cycles in the present experiment, the internal pores were never completely dry or wet; thus, the comprehensive ability of the oxygen supply and diffusion was better than that of those of the fully dry and fully moist states.

**Figure 16:**
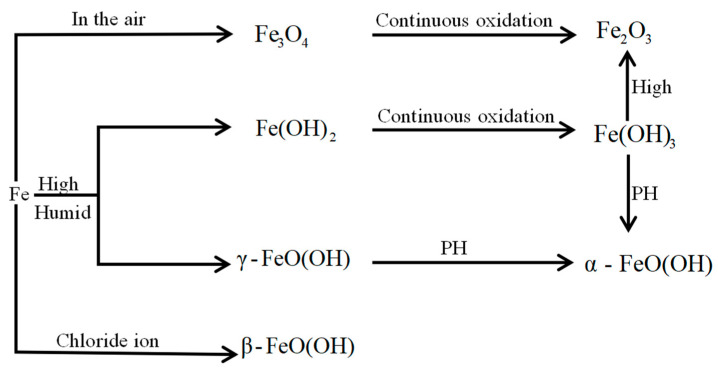
Transformation process of the main oxidation products of iron.

When the rust expansion cracks penetrate the concrete cover, the steel bars inside the concrete become oxygen-enriched. In such an oxygen-enriched environment, the rebars produce more high-valence corrosion products, such as γ-FeO(OH) during the dry–wet cycle (see Figure 16).

On the basis of the composition and contents of the eight samples and the transformation process of the iron oxidation products, it was concluded that when the dry-to-wet ratio was reduced, the γ-FeO(OH) and β-FeO(OH) contents increased and the α-FeO(OH) content decreased. When the other conditions were identical, reducing the dry-to-wet ratio lengthened the corrosion cracking time and facilitated the formation of β-FeO(OH) and its intermediate product γ-FeO(OH) through the large impact of chloride ions.

## 6. Conclusions

The main conclusions of this paper are summarized as follows:

(1) A theoretical model of nonuniform corrosion cracking of steel bars was proposed. In this model, when the maximum filling thickness of the corrosion products exceeds the radial displacement of concrete, the concrete cracks.

(2) An analysis of the relationship between the nonuniformity coefficient and reliability index demonstrated that the larger the nonuniformity coefficient, the smaller the reliability index. The reliability index decreased most rapidly in the range of 0 ≤ *K* ≤ 1.

(3) The development mode of the rust pits on the surfaces of the steel bars did not depend on the wet–dry cycling environment of the concrete.

(4) The Fe and O distributions in the energy spectrum of the corroded reinforced concrete were divisible into five areas: reinforcement, rust layer, oxide skin, polluted concrete, and unpolluted concrete.

(5) Based on the corrosion principle of reinforced concrete and the XRD results, the main corrosion products that were under the different dry and wet cycles were α-FeO(OH), β-FeO(OH), γ-FeO(OH), Fe_2_O_3_, and Fe_3_O_4_. The further comparative analysis indicated that a reduction in the dry–wet ratio of the dry–wet cycles increased the γ-FeO(OH) and β-FeO(OH) contents.

## Figures and Tables

**Table 1:** Averages and standard deviations of concrete parameters.

Parameter	*f_c_* (MPa)	*c* (mm)	*d* (mm)	*ρ*
Average value	28.66	33.8	32	0.806
Standard deviation	5.52	13.1	0.79	250.23
Distribution type	Normal distribution	Normal distribution	Normal distribution	Normal distribution

**Table 2:** Mix proportions of the test concrete.

Water–Cement Ratio	Water (kg)	Cement (kg)	Sand (kg)	Strength Grade of Concrete
0.6	250	417	1734	C30

**Table 3:** Test groups.

NaCl Concentration (%)	*d* (mm)	*l* (mm)	*h* (mm)	Soaking Time (h)	Drying Time (h)	Number of Specimens
7	8	32	100	12	12	5
				16	8	5
		40		16	8	5
				20	4	5
	10	32		12	12	5
				16	8	5
		40		16	8	5
				20	4	5

*d* = diameter of steel bars; *l* = specimen diameter; *h* = specimen length.

**Table 4:** Cracking time of concrete covers.

Sample Number	Crack Width (mm)	Cracking Time (h)	Sample Number	Crack Width (mm)	Cracking Time (h)
32-8-12-1	0.14	986	40-8-16-1	0.10	1586
32-8-12-2	0.12	1004	40-8-16-2	0.14	1472
32-8-12-3	0.11	1076	40-8-16-3	0.12	1547
32-8-12-4	0.18	975	40-8-16-4	0.10	1483
32-8-12-5	0.10	897	40-8-16-5	0.10	1583
32-8-16-1	0.14	1091	40-8-20-1	0.12	1765
32-8-16-2	0.13	1120	40-8-20-2	0.10	1849
32-8-16-3	0.16	1210	40-8-20-3	0.10	1776
32-8-16-4	0.10	1147	40-8-20-4	0.14	1869
32-8-16-5	0.14	1247	40-8-20-5	0.16	1669
32-10-12-1	0.18	1254	40-10-16-1	0.12	1686
32-10-12-2	0.20	1470	40-10-16-2	0.18	1784
32-10-12-3	0.14	1386	40-10-16-3	0.10	1625
32-10-12-4	0.21	1184	40-10-16-4	0.16	1759
32-10-12-5	0.14	1243	40-10-16-5	0.14	1659
32-10-16-1	0.12	1480	40-10-20-1	0.20	1840
32-10-16-2	0.14	1577	40-10-20-2	0.16	1948
32-10-16-3	0.10	1475	40-10-20-3	0.10	2076
32-10-16-4	0.13	1355	40-10-20-4	0.14	1929
32-10-16-5	0.14	1545	40-10-20-5	0.10	1879

## Data Availability

The data used to support the findings of this study are available from the corresponding author upon request.
